# A Different Diabetes: Arsenic Plus High-Fat Diet Yields an Unusual Diabetes Phenotype in Mice

**DOI:** 10.1289/ehp.119-a354b

**Published:** 2011-08-01

**Authors:** Julia R. Barrett

**Affiliations:** Julia R. Barrett, MS, ELS, a Madison, WI–based science writer and editor, has written for *EHP* since 1996. She is a member of the National Association of Science Writers and the Board of Editors in the Life Sciences.

Obesity is the leading cause of type 2 diabetes mellitus. Because inorganic arsenic has been associated with type 2 diabetes in laboratory studies, chronic arsenic exposure has been hypothesized to heighten the risk of diabetes in obese individuals. A new study reveals a synergistic relationship between inorganic arsenic exposure and obesity that impairs glucose tolerance in mice **[*EHP* 119(8):1104–1109; Paul et al**.].

From age 4 weeks, male C57BL/6 mice, a strain susceptible to developing diet-induced diabetes, ate either a low- or high-fat diet and drank purified water (controls) or water with inorganic arsenic at 25 or 50 ppm. After 20 weeks, an oral glucose tolerance test was used to assess glucose homeostasis in the mice, and serum insulin was measured before and after glucose administration. Following sacrifice 7–10 days later, serum and tissue samples were collected to determine hydration, serum and hepatic triacylglycerol content, and body distribution of arsenic and arsenic metabolites. Statistical analysis focused on effects of diet, inorganic arsenic, and interactions between the two on the development of diabetes.

As expected, control mice on the high-fat diet weighed more and accumulated more fat than control mice on the low-fat diet. They exhibited higher fasting blood glucose, higher fasting and post–glucose challenge serum insulin, and greater insulin resistance, symptoms consistent with type 2 diabetes. In contrast, arsenic-exposed mice on the high-fat diet accumulated less fat than unexposed mice on the same diet and had lower serum triacylglycerol, fasting blood glucose, and insulin measures. However, glucose intolerance was high in these mice, even exceeding that observed in controls on the same diet.

The results suggest inorganic arsenic reduces diet-induced obesity in mice but acts synergistically with a high-fat diet to produce glucose intolerance, a symptom typically associated with prediabetes. The combination of glucose intolerance and elevated blood glucose levels with near-normal plasma insulin levels differs from the typical phenotype for type 2 diabetes.

The authors point out that the measures indicating near-normal insulin sensitivity, when paired with glucose intolerance, can reflect impaired insulin production by pancreatic ® cells—a characteristic of advanced diabetes. However, the study did not measure ® cell function and signals that regulate it and thus cannot offer insight on this possibility. More comprehensive investigation is needed to determine how inorganic arsenic exposure, alone and with a high-fat diet, may induce type 2 diabetes.

